# A comparison of conventional rapid methods in diagnosis of superficial and cutaneous mycoses based on KOH, Chicago sky blue 6B and calcofluor white stains

**Published:** 2018-12

**Authors:** Parvaneh Afshar, Laleh Vahedi Larijani, Hamed Rouhanizadeh

**Affiliations:** 1 Research and Development Unit of Referral Laboratory, Mazandaran University of Medical Sciences, Sari, Iran; 2 Department of Pathology, School of Medicine, Mazandaran University of Medical Sciences, Sari, Iran; 3 Gastrointestinal Cancer Research Center, Mazandaran University of Medical Sciences, Sari, Iran; 4 Department of Pediatrics, School of Medicine and Deputy of Health Management, Mazandaran University of Medical Sciences, Sari, Iran

**Keywords:** Rapid diagnosis, Dermatomycosis, Potassium hydroxide, Chicago sky blue 6B, Calcofluor white

## Abstract

**Background and Objectives:**

Rapid confirmation of dermatomycoses is desirable, as it allows the clinicians to initiate appropriate therapy immediately. In this study, the utility of a novel contrast stain, Chicago sky blue stain, was compared with potassium hydroxide mount and calcofluor white stain to determine the causative fungal elements in the rapid detection method.

**Materials and Methods:**

In this survey, 189 samples of suspected dermatomycosis infections were assessed in 3 incubation times of 30 minutes, 2 hours, and > 6 hours.

**Results:**

Positive cases were shown in Chicago sky blue 6B (55%), calcofluor white (53.4%), and potassium hydroxide (36%), with 30-minute incubation. Positive results increased in other incubation times. Sensitivity, specificity, PPV, NPV and accuracy of Chicago sky blue 6B were 97%, 100%, 100%, 96% and 98% and, for potassium hydroxide, they were 66%, 98%, 97%, 98%, 80% versus CFW, respectively.

**Conclusion:**

This study provides evidence that the Chicago sky blue 6B stain is a simple, fast and cost-effective method.

## INTRODUCTION

Mycotic infections, which depend on specific geographical and climatic areas, lifestyle, patient age, occupation, migration, sport activities, and drug therapy, are very common infections of skin, hair, and nails in many countries (1, 2). They are the fourth most common cause of health care problems affecting millions of people worldwide-especially in the pediatric group (2). Superficial fungal infections have become a major cause of morbidity and mortality in clinically debilitated or immune compromised patients (3).

Major etiological agents of dermatomycoses include dermatophytes and yeasts (*Candida* spp. and *Malassezia* spp.) (4). Diagnosis of superficial mycosis is often clinically established; however, laboratory confirmation is required for more difficult and atypical lesions and for type determination of causative fungi. Laboratory diagnostic procedures in dermatological mycology are based on direct microscopy and culture. Potassium hydroxide (KOH) wet mount preparation used for direct microscopy is generally considered as conventional rapid test (5, 6).

The fungal cell is surrounded via a cell wall, which is essential to maintain the cell shape and regulation of the absorption of substances from the environment. The cell wall structure is dynamic and can match different physiological conditions or environmental states (7). Fungally specific enzymes that manufacture the cell wall are attractive targets for antifungal therapies. In particular, the cell wall-targeting class of antifungal drugs, chitin, glucans, and mannoproteins are the major polysaccharide components that are present in cell wall of most fungi (8, 9).

Potassium hydroxide is a keratin digestion reagent that will dissolve proteins, lipids, and lyse epithelium. The fungus element will withstand the KOH solution (10%–30%), because it contains chitin and glycoproteins in the cell wall. KOH determines fungal elements between keratin cells quickly and irreversibly without staining particular specimens. This clearing agent provides a significant difference in brightness between fungal cells and the sample background and helps to improve quality of results (10).

Calcofluor white (CFW) is a fluorescent stain for rapid detection of yeasts, fungi, and parasitic organisms, and it is a disodium salt of 4, 4′-bis (4 anilino-bis-diethylamino-5-triazin-2-ylamino) 2, 2′-stilbene-disulfonic acid. The non-specific fluorochrome stain is a specific dye for chitin and cellulose with the ability to show β (1–3) and β (1, 4)-linked polysaccharides and display fluorescence when exposed to long wavelength ultraviolet and short wavelength visible light (11). CFW also stains other tissue elements as collagen, keratin, and elastin. CFW is used on all types of samples, such as fresh, fixed, frozen, tissue paraffin-embedded, and clinical specimens (11, 12).

Chicago Sky Blue 6B (CSB) is a large organic acid and diazo dye tetrasodium 6, 60-{(3, 30-dimethoxy [1, 10-bipheyl]-4, 40-diyl) bis (azo)} bis {4-amino-5-hydroxynaphthalene-1, 3-disulfonate}, also known as Atlantic resin fast blue. This dye can stain the fungal elements (blue) and the background tissue (pink) in contrasting colors structurally related to glutamate, which is a potent and efficient competitive inhibitor of vesicular glutamate uptake (2).

Thus, early diagnosis of a fungal infection and differentiating the fungal elements from other etiological agents, such as particular bacterial cells that produce almost similar symptoms, are highly important, as physicians can have the needed information to start the appropriate therapy earlier (13). Therefore, this study aimed to compare the diagnostic efficacy of a direct microscopic evaluation smear based on KOH (Reference method), CFW (Gold standard), and CSB staining in 3 time points of 30 minutes, 2 hours, and >6 hours (overnight) after smear preparation. Then, sensitivity, specificity, positive predictive values (PPV), negative predictive values (NPV), and accuracy between techniques were evaluated to describe a fast and reliable protocol.

## MATERIALS AND METHODS

### Samples collection.

**Fig. 1.**
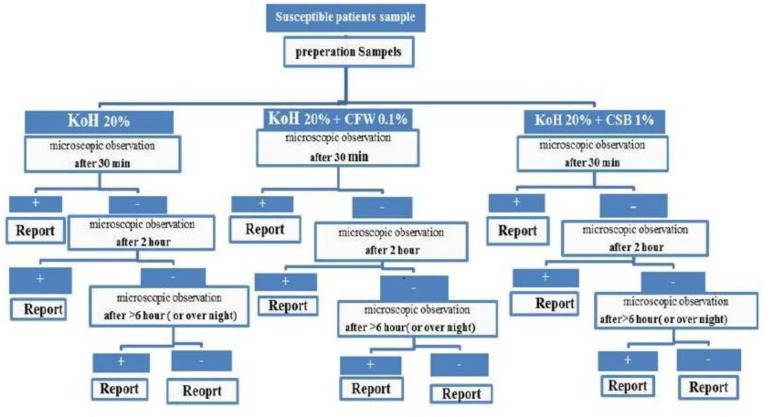
Sample processing and analysis flow chart

In a cross sectional study, specimens were collected from 189 patients younger than 20 years who were suspected of having dermatomycosis infection after scraping their infected area using a sterile scalpel blade. The ethics committee of Mazandaran University of Medical Science approved this study. Also, a written informed consent was obtained from each participant prior to enrollment. The clinical materials were then evenly divided for KOH preparations, CSB-KOH dye, and CFW-KOH staining. All samples of each technique were evaluated at 3 time points of 30 minutes, 2 hours, and > 6 hours (overnight) in the Department of Research and Development of Referral Laboratory, Mazandaran University of Medical Sciences (RLMUMS) (Fig. 1). Exclusion criteria were atypical cases and patients on treatment with systemic and/or local corticosteroid agents and/or antifungal drugs at least 1 month prior to mycological examination. Sampling continued to the point that the total positive cases were almost equal to 100 in KOH preparations (Reference method) in > 6 hours (overnight) review.

### Laboratory procedures: Preparation of the KOH 20% aqueous solution W/V.

KOH working solution was prepared by dissolving 2 g of the potassium hydroxide crystal (105032 Merck, Germany) in 8 mL of deionized water and 2 mL of glycerol forming 20% solution. A drop (50 μL) of this solution was placed onto a microscope slide containing the sample. The specimen was given a short heat and was then evaluated microscopically for the presence of fungal elements.

### Preparation of CSB stain 1% aqueous solution W/V.

Working solution of CSB stain was prepared by dissolving 0.1 g of the CSB 6B dye (C8679 Sigma, USA) in 10 mL of deionized water forming 1% solution and was kept in a brown dropper bottle at room temperature. A drop (20–25 μL) of this solution was mixed with a drop of 20% KOH and was then placed on a microscope slide (V/V), which contained the sample.

### Preparation of CfW stain 0.1% aqueous solution W/V.

Working solution of CFW stain was prepared by dissolving 0.01 g of the CFW dye (18909 Fluka, Canada) in 10 mL of deionized water forming 0.1% solution and was kept in a brown dropper bottle at room temperature. A KOH/CFW solution V/V (a drop 20–25 μL of CFW work solution was mixed with a drop of 20% potassium hydroxide) can help to identify the fungus in the tissue under ultraviolet illumination. These reagents were mixed and placed onto a microscope slide containing the sample.

**Fig. 2.**
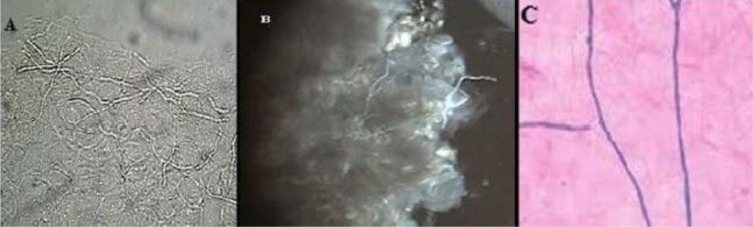
Fungal elements in skin scraping stained at ×40 magnification; A: KOH, B: CFW-KOH, C: CSB-KOH

Fungal elements were detectable in KOH, CFW-KOH, and CSB-KOH preparation via green, white, and blue colors, respectively (Fig. 2). The fungal element cell walls stained blue against the purplish background of cellular debris by CSB stain and were easily discerned even on scanner view (≤10 × magnification) compared to the KOH and/or CFW mount. On higher magnification (≥ 10 ×), hyphae or yeasts cells were observed, with the characteristic of true hyphae, psoudohyphae, budding cells, and spaghetti & meatball appearance. Darker staining was observed when CSB stain was kept more than 30 minutes.

### Consuming materials' safety information.

Safety information sheets contain details on the composition, safety, handling, storage, disposal, and transportation of materials, and other regulatory information. They are routinely used in performing risk assessments for chemicals or other products used in the operating environment, in compliance with CSSH (Control of Substances Hazardous to Health) regulations (10). According to safety information, CSB, CFW, and KOH are reported to have a corrosive effect on metals and skin and to seriously damage the eyes or respiratory tract. Therefore, it is recommended to use protective gloves, eye shield, and dust respirator filter during handling and working with the material. However, low concentrations were used in this study, which is unlikely to cause any significant problems.

### Reagents quality control.

A positive quality control was performed by staining any of the fungus cultures, such as a common mold, *Candida*, and *Tinea* spp. (14, 15), in a referral laboratory in Mazandaran University of Medical Sciences on a glass slide. Fungal elements should appear in blue and white color in CSB and CFW preparations and lack color or have pale green color in KOH mounts, respectively. For negative quality control alone, a drop of reagents was put on the slide, which by itself should be free of any germs and particles.

### Statistical analysis.

Statistical analysis was performed by the IBM SPSS Statistics software version 21, followed by McNemar test for comparing paired tests. In addition, variable intergroup comparison was analyzed using Manne-Whitney test. Sensitivity, specificity, PPV, NPV, and accuracy were calculated according to standard equations (16–18). To compare these diagnostic methods, CSB stain and KOH mount were used as the reference method, as they are the most commonly used and practical tests available for the diagnosis of dermatomycoses in most diagnostic laboratories (general and non-researching centers), using CFW preparation (gold. St). Also, P < 0.05 was considered statistically significant.

## RESULTS

A total of 189 patients younger than 20 years were included in the study; of them, 112 (57.2%) were male and 77 (42.8%) were female. The mean age was 12.23 ± 5.17 years. Among 120 positive dermatomycoses cases, 66% were male and 33% were female.

**Fig. 3.**
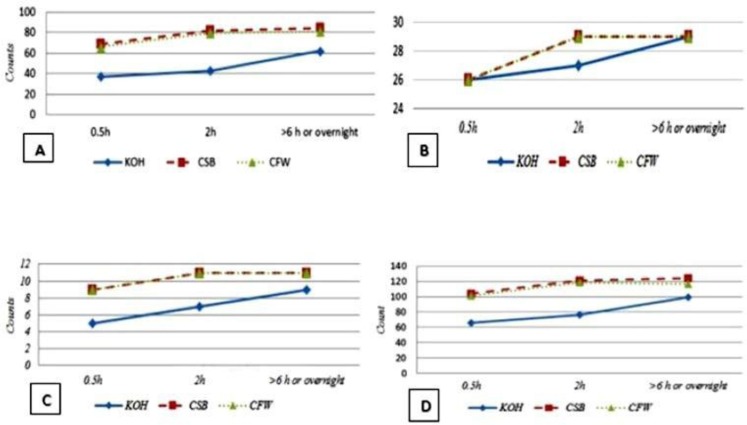
Relation between positive samples and incubation time of diagnosis; A: Dermatophyte, B: P. Versicolor, C: Candida, D: Total

The results of comparing conventional microscopy techniques of CSB and KOH, as a reference method according to CFW stain of fungal elements diagnosis, are demonstrated in Tables 1, 2 and Fig. 3. Out of 189 patients, in direct microscopy with KOH, CFW, and CSB mounts, positive results were shown in 36%, 53.4% and 55% of the samples, with a 30-minute incubation, respectively. The most diagnostic difference was observed in the positive rates of dermatophytes fungal elements, which were 19.6% in KOH preparation, 34.4% in CFW, and 36.5% in CSB, with 30-minute incubation; this rate was 22.8%, 41.8% and 43.4% in 2-hour incubation and 32.8%, 42.9%, and 44.4% in >6 hours (overnight) incubation, respectively (Fig. 3). Statistical analysis on sensitivity, specificity, PPV, NPV, and overall accuracy of CSB and KOH stains, using CFW preparation as the standard method, are summarized in Table 3.

**Table 1.** Frequency fungal elements recognized based on rapid direct examination methods and incubation time (N = 189)

	**30 minutes N (%)**		**2-hour N (%)**		**>6 hour or overnight N (%)**	

	**Positive**	**Negative**	**Positive**	**Negative**	**Positive**	**Negative**
**^a^KOH**	68 (35.98)	121 (64.02)	77 (40.74)	112 (59.26)	100 (52.91)	89 (47.09)
**^b^CFW**	101 (53.44)	88 (46.56)	119 (62.96)	70 (37.04)	121 (64.02)	68 (35.98)
**CSB**	104 (55.03)	85 (44.97)	122 (64.55)	67 (35.45)	124 (65.61)	65 (34.39)

a KOH (Reference method)

b CFW (Gold. Standard)

**Table 2.** Frequency positive fungal elements recognize based on rapid direct examination and incubation time (N= 189)

**Test**	**30 minutes N (%)**			**2-hour N (%)**			**>6 hour or overnight N (%)**		

	Dermatophyte	Non dermatophyte		Dermatophyte	Non dermatophyte		Dermatophyte	Non dermatophyte	
		
		*Candid*	*Malassezia*		*Candida*	*Malassezia*		*Candid*	*Malassezia*
KOH	37 (19.6)	31 (19.4)		43 (22.8)	34 (18)		62 (32.8)	38 (20.1)	
		5 (2.6)	26 (13.8)		7 (3.7)	27 (14.3)		9 (4.8)	29 (15.3)

CFW	66 (34.9)	35 (18.6)		79 (41.8)	40 (21.1)		81 (42.9)	40 (21.1)	
		9 (4.8)	26 (13.8)		11 (5.8)	29 (15.3)		11 (5.8)	29 (15.3)

CSB	69 (36.5)	35 (18.6)		82 (43.4)	40 (21.1)		84 (44.4)	40 (21.1)	
		9 (4.8)	26 (13.8)		11 (5.8)	29 (15.3)		11 (5.8)	29 (15.3)

**Table 3.** Comparison of CSB and KOH as reference method according to CFW stain as Gold.St method for diagnosis of fungal elements in 30 minutes incubation (N= 189)

**Test**	**Positive Cases N (%)**	**Sensitivity (%)**	**Specificity (%)**	**PPV^a^ (%)**	**NPV^b^ (%)**	**Accuracy (%)**
KOH	68 (36)	66	98	97	98	80
CSB	104 (55)	97	100	100	100	98

a Positive predictive values (PPV)

b Negative predictive values (NPV)

## DISCUSSION

Immediate diagnosis and therapy of dermatomycosis is made based on disease history and clinical examination and can be supported by traditionally direct wet preparation examination, with the common use of potassium hydroxide (KOH) solution (10). Besides KOH, various contrast dyes and clearing agents are available for fungal element recognition, such as NaOH, SDS, Parker ink, Blue-Black ink, Chlorazol Black E, CFW Cotton Blue C4B, and CSB (10, 19). KOH is an inexpensive reference method that does not create color distinction and utilization. However, visualizing fungal parasites in very poor-quality specimens was highly difficult, thus, it would yield considerable proficiency in exam interpretation (20, 21). Therefore, to decrease these limitations, special stains, such as CFW and CSB, have been utilized and most of the time it was not possible to test the stain concentrations. Thus, to verify the accuracy of this technique, result recheck is required with validation methods (2). These colors can create a good color contrast in *Malassezia, Candida*, and filamentous fungi, particularly dermatophytes, leading to a comfortable and accurate fungal elements detection with a simple method assessment (22).

Calcofluor does not have any health and safety implications when used in the laboratory. However, a previous survey has shown the added whitener compounds to KOH preparations that led to improving the detection of fungal elements via increasing the background debris contrast, but for this, expensive equipment (immunofluorescence microscope) are needed (22). CSB stain is a quick, safe, simple, repeatable, and non-aggressive procedure, which does not need expensive equipment. Also, susceptible analytical errors are less than other direct exams in this method (19). CFW and CSB stains are particularly bound to the chitin, a β (1, 4)-linked homopolymer of N-acetyl glucosamine fungal cell wall, which allows an easy differentiation from other artifacts, such as epithelial cell borders (mosaic fungus).

The number of positive cases in >6 hours (overnight) incubation in all 3 preparation methods was more than 2 hours, and 30 minutes incubation. The number of positive cases in CFW-especially CSB- at 30-minute incubation was more than KOH method in about > 6 hours (overnight) incubation, which indicates the accuracy of the report (Table 1–3), similar to results of other studies (23, 24). In this survey, comparing CSB dye with another direct microscopy fungal identification has shown excellent agreement with the highest positivity detection rates relative to KOH and even CFW (Table 1–3). The difference in the final report of the evaluation at various incubation times is perhaps due to the slow absorption of CSB by some types of fungi (2, 10, 20). Therefore, when identifying fungi elements in direct observation, false negative results should be removed and reassessment must be done in the next day-particularly in all negative report samples.

The sample assessments of about 30-minute incubation stage with 104 positive cases in CSB stain panel showed high sensitivity (97%) and specificity (100%), whereas conventional KOH preparation showed 66% sensitivity and 98% specificity only with 68 positive cases (Table 3). However, final diagnosis and distinction dermatomycoses infection-especially dermatophytes-increased with an increase in incubation time (2, 24, 25).

Given the fact that the biggest drawback of the culture-based approach is the slow growth of fungal elements, an incubation time of 3–4 weeks for dermatophytes is required for the final report. False negative results can be increased in culture method via the followings: viable but noncultureable (VBNC) or non-viable elements; the small number of microorganisms; copathogenic or contamination overload of microorganisms; and unsuitability of media, incubation time, transport, and temperature time (13, 26, 27). Unlike most studies that used the culture method as the gold standard, the evaluation scale was used in CFW study methods as a rapid laboratory recognized method, and this was the main reason for disagreement in sensitivity and specificity results (2, 24, 25).

Mutations in glucan synthase genes reduce glucan levels in the cell wall while stimulating release pathways, leading to increased chitin synthesis (The pathway restores the strength of the cell wall matrix and prevents antifungal action.) (28). It seems that more positive dermatophytes (3 cases) have been reported in CSB compared to Gold.St CFW method in > 6-hour (overnight) incubation, which might have been due to changes of fungi cell wall chitin level after taking anti-fungal drugs, the target point for CFW stain with none or less fluorescent color. Identifying dermatomycoses is considered an important challenge for experts (22, 29). However, many other studies have recommended that CSB solution, by staining fungal elements, can be used as an excellent alternative solution to eliminate direct diagnostic problems-especially in skin fungi (2, 21). On the other hand, molecular techniques using DNA probes for *in situ* hybridization and polymerase chain reaction have demonstrated higher degrees of sensitivity, specificity, accuracy, and precision. However, as utilizing this method requires expensive equipment and takes up to 2–3 days to finish, (30) it is suggested that this method be done using molecular techniques and other methods.

## CONCLUSION

The false-negative dermatomycoses reporting rate decreased in the approximately > 6-hour (overnight) incubation time. Considering the fact that there are many different samples in general diagnostic laboratory centers, the data in this survey recommend that suspicious fungal samples be prepared with CSB and evaluated at least by approximately 2-hour incubation in room temperature-particularly in the dermatophyte agents. In addition to increasing the reported accuracy, the results will be presented to the patient and physicians in a shorter time.

On the other hand, with increased efficiency, precise diagnosis, and less cost (21), the estimated retail price for each 10 mL bottle CSB stain 1%, which stains almost 400 specimens, is about 200 000 IRR ~ $2.00 (US). This will reduce the additional costs of prescribing a wrong drug by the physician and its complications for the patient due to laboratory misdiagnosis of the disease. Also, using an appropriate method may be useful in developing educational and preventive strategies and consequently reducing health care expenditure.
